# Young internal migrants’ major health issues and health seeking barriers in Shanghai, China: a qualitative study

**DOI:** 10.1186/s12889-019-6661-0

**Published:** 2019-03-22

**Authors:** Chunyan Yu, Chaohua Lou, Yan Cheng, Yuanqi Cui, Qiguo Lian, Ziliang Wang, Ersheng Gao, Ling Wang

**Affiliations:** 1 School of Public Health, Key Laboratory of Public Health Safety, Ministry of Education, Fudan University, NO.130 Dong’an Road, Xuhui District, Shanghai, 200032 People’s Republic of China; 2 NHC Key Lab. of Reproduction Regulation (Shanghai Institute of Planned Parenthood Research), Fudan University, NO.779 Old Humin Road, Xuhui District, Shanghai, 200237 People’s Republic of China; 3 Family Planning NSW, 328–336 Liverpool Road, Ashfield, NSW 2131 Australia; 4 Shanghai Municipal Health Commission, NO.300 Village Shibo Road, Pudong District, Shanghai, 200125 People’s Republic of China

**Keywords:** Internal migrant youth, Qualitative study, Thematic analysis, Health equity, Health seeking barriers

## Abstract

**Background:**

China is experiencing a sizeable rural-urban flow, which may influence the health of internal migrant youth deeply. Disadvantages in the city are highly likely to contribute to health issues among the young internal migrant population. The current qualitative study is to explore how internal migrant young people view the health issues they face, and the services and opportunities they could seek in their host community.

**Methods:**

Data were collected from 90 internal migrant youth aged between 15 and 19 years old and 20 adult service providers who worked with them in a community of Shanghai, where the population of internal migrants was relatively large. Four types of qualitative research methods were used, including key informant interviews with adults, in-depth interviews with adolescents, a photovoice activity with adolescents and community mapping & focus group discussions with adolescents. Guided by the ecological systems framework and the acculturation theory, thematic analysis was conducted using ATLAS.Ti 7.0 software.

**Results:**

While younger migrants had a limited understanding of health, elder migrant youths were more sensitive to societal and political factors related to their health. Mental health and health risk behaviors such as smoking, violence and premarital unsafe sex were thought as major health issues. Internal migrant youths rarely seek health information and services initiatively from formal sources. They believed their health concerns weren’t as pressing as the pressure coming from the high cost of living, the experience of being unfairly treated and the lack of opportunities. Participants also cited lack of family and social support, lack of awareness and supportive policies to get access to community and public services as impacting health factors.

**Conclusions:**

The study’s findings provide the insight to the social contexts which influence the health experience, health seeking behaviors, and city adaptation of young internal migrants in their host community. This research stresses the importance of understanding social networks and structural barriers faced by migrant youth in vulnerable environments. A multidimensional social support is essential for internal migrant youth facing present and potential health risks.

**Electronic supplementary material:**

The online version of this article (10.1186/s12889-019-6661-0) contains supplementary material, which is available to authorized users.

## Background

Since late twentieth Century, rates of urbanization have increased dramatically worldwide, particularly in developing countries. This phenomenon is particularly apparent in China, where much of the expansion is due to internal rural-to-urban migration. Those who have left their hometowns to live and work elsewhere are defined as internal migrants (also called “the floating population” or “liudong renkou”) [1]. According to the data from the sixth national census, the number of internal migrants in China had reached 221 million in 2010, accounting for 16.5% of the total population [1]. Half of the internal migrants were among the age of 15 to 30. The number of registered ones in Shanghai (the largest city in China with a population of approximately 23 million in 2010) alone was about 8.98 million (accounting for 39% of the city’s population) [1]. Though urbanization and migration were seen as a strategy for poverty alleviation by many internal migrants, their health and development were drastically influenced [2]. For example, the vaccination rates among internal migrant children and adolescents are reported to be lower than the average among their urban peers [3]. There were more reproductive health problems among young internal migrants. Rural-to-urban migrant males were significantly less likely to use condoms at first sex and consistent contraceptive use with first partner compared with nonimmigrants [4].

Adolescence and young adulthood represent crucial life stages. Age 15~19 is a turning point as the youth begin to navigate their unique ways through societal structures. It is the time they shape ideas about their talents and make aspirations for the future [5]. At these pivotal life stages, young migrants may experience unique stressors. Many are away from family support and their previous social networks for the first time, with their personal identities and sense of selves developed. It is conceivable that certain health risk factors are related to migration: the unpleasant and unfamiliar working and living environment, new and challenging socio-cultural-political atmosphere, harsh contexts of urban integration and institutional barriers to health-protective services including healthcare delivery [6–8]. Persistent disadvantages in the city are highly likely to contribute to health issues among the young migrant population [9–11]. Studies that focus on the mental health status of Chinese internal migrants showed that their disadvantages, such as lower self-esteem, higher loneliness, higher social anxiety, and more perceived discrimination, may cause physical functional disturbance or psychological distress and behavioral problems [12–14]. For migrant girls who started to earn a living early, some would experience a higher likelihood of entering sex trade [15].

Hukou, the Chinese system of household registration, was initially designed for the restriction on the geographical mobility of the population in 1958. Each Chinese resident was assigned to a fixed place of residence. Rural or urban residents were not allowed to move to other cities [16, 17]. This situation has dramatically changed after the 1978 economic reforms. As the urban economy was developing rapidly, the government relaxed the migration policies and allowed the rural citizens to move to the cities for employment. Although there were channels (include the pursuit of higher education and then employment in government, or influential family connections, etc.) for Hukou mobility allowing some rural Hukou holders to acquire urban Hukou, the majority of migrants remained to have their Hukou registered in their hometown [16]. Furthermore, because of Hukou system, migrants who are not registered in their new place of residence cannot access certain state-level entitlement and social benefits. Hukou system is regarded as an important institutional barrier, particularly for rural-to-urban migrants, to the achievement of equal rights to employment, schooling, housing, health care, and social services [18].

While internal migrants were frequently represented as victims who deserve compassion and protection in the media, mixed views towards them existed, such as being accused of causing problems and threats to the community. It was reported that in 2000 more than 50% of criminal suspects in big cities like Shanghai, Beijing and Guangzhou were migrant workers [19]. Meantime, studies on internal migrant workers in China showed that the second-generation migrants were different from their parental generation. They were better off than their parents in terms of education and health status but still, lack supporting system [17]. Several surveys reported that social support from different types of relationships was deferentially related to better health [12, 20, 21]. It remains unknown why and by which mechanism the domains of supports could provide the benefit for the health of internal migrant youth [22]. Besides, the problems that internal migrants encountered and the solutions to control or transform them have been constructed by the elites and rarely by migrant themselves. Though WHO defined health as a “state of complete physical, mental, and social well-being, and not merely the absence of disease or infirmity” [23], little research exists on Chinese internal migrant youths’ perception about what they think health is, what their major health issues are, and how they think of the health care services [9]. Thus, the current qualitative study is to explore how internal migrant young people define health, what are their major health issues, and how they think of the health services and social adaptation related opportunities they could seek in their host community.

### Conceptual framework

The study used Bronfenbrenner’s ecological systems theory as the primary framework and acculturation theory as supplementary to analyze the health issues and health-seeking behaviors of migrants. The ecological systems framework operates under the assumption that individual actions are a result of multiple, interacting levels – the microsystem, exosystem, and macrosystem [24]. The microsystem is the immediate environment in which the individual lives including home and families. The exosystem is the community in which the microsystem is situated. In a migrants’ life, this may mean the neighborhood, workplace, school or community. The macrosystem level factors are overarching cultural norms, traditional values, and policies that guide social processes. In our paper, we consider health issues and health-seeking behaviors a result of a complex network of environments and being influenced by micro (such as personal incomes, awareness of health knowledges, etc.), exosystem (e.g., influences from peers, colleagues and communities) and macro-level (e.g., Hukou policies, opportunities, etc.) forces. Additionally, according to acculturation theory [25], contact and reciprocal influence with a dominant culture for migrants in host city will result in challenges and changes in this group to adapt to new attitudes, knowledge, and behaviors [26]. During the process, comparing to locals, the individual may need to deal with extra acculturative stressors such as social distance and discriminant attitudes, which may bring negative effects on well-being. Acculturation of internal migrants unfolds as a process and is situated in an ecological context [27]. Within individual and family domain, emotional instability, and the discrepancies between family members could exacerbate acculturation outcomes while parental connectedness and positive coping reactions can buffer the negative effects on psychological well-being. At the interpersonal level, school and workplace exert significant influences on the student migrant and labor migrants, respectively. At the societal level, opportunities, attitudes, policies and prejudice affect the acculturation experiences of internal migrants and influence their psychological and socio-cultural adaptation [28].

## Methods

### Study design

The Well-being of Adolescents in Vulnerable Environments (WAVE) was a global study of five-city (Baltimore, USA; Ibadan, Nigeria; Johannesburg, South Africa; New Delhi, India; and Shanghai, China.) young people in vulnerable environments with the goal of discovering ways to connect youth to health [29]. The study was designed to understand the factors that facilitate and hinder disadvantaged adolescents from obtaining the health information and services they need to secure good health. This paper is based on the qualitative data from Shanghai site only.

The study was conducted in a suburban community where migrants lived concentratedly from September to December 2011. Four types of qualitative methods were employed: one method with adults --key informant interviews (KIIs), and three methods with adolescents -- in-depth interviews (IDIs), community mapping & focus group discussions (CMGs), and photovoice methods (PVs). The field guide of each activity was uploaded as the supplementary files. The purpose of KIIs was to ask adults who had contacts with internal migrant youth in the community about what they considered to be the biggest health challenges faced by migrant adolescents, and the factors contributed to their health challenges (See Additional file 1: KIIs Guide). Young participants were only recruited for one type of research methodology, allowing us to get a broader range of adolescent perspectives. The IDIs were conducted to explore how internal migrant adolescents perceived their own health, what they considered as their primary health challenges and factors contributed to those challenges, as well as how they sought help for solving problems (See Additional file 2: IDIs Guide). Other than conducting interviews with adults and adolescents, CMG and PV exercises, which used participatory action research approaches, were also included to strengthen our ability to triangulate the findings. The CMG activity was a day-long activity for a group of youth that included a mapping exercise and focus group discussion to highlight issues relevant to yong internal migrants’ health, available resources, and sources of support as well as the health risk that existed in their community (See Additional file 3: CMG Guide). The PV exercise actually means giving a ‘voice’ to participants, which allows them to explore issues and topics visually and verbally in a way that may be more effective than by using words alone [30]. For this exercise, young people were trained in the art of photography, and during the following days, took photos that capture what they believed is the meaning of health in their community. Then, migrant youth explained their pictures through captions and discussions during that week (See Additional file 4: PV Guide). The methodology has been described in detail in “Adolescents’ perceptions of health from disadvantaged urban communities: Findings from the WAVE study” [29].

### Participants

Twenty-three (13 male and 10 female) adolescents were in-depth interviewed face-to-face with an average duration of 50 min. Eight adolescents (3 male and 5 female) were recruited to participate in four days’ photovoice activity, where adolescents were trained in photography and took photos of what they perceived as the meaning of *health* in their communities. Fifty-nine adolescents were invited to participate in the half-day community mapping and focus group discussions, where adolescents were organized in eight groups based on age and gender and were asked first to draw maps of their communities and then to discuss issues related to their community maps. Adolescents were aged 15~19 years old, living in the study community but without *Hukou* of Shanghai. Most of these adolescents were recruited with the help of key informants; others were recruited by their friends’ referral. Ninety adolescents and twenty adults in total finally participated in the study in Shanghai site. (Table 1).

**Table 1:** Description of the sample

Migrant Youth’s Activity				
		IDI %(n)	PV %(n)	CMG %(n)
Age	15–17	52.2(12)	62.5(5)	40.7(24)
	18–19	47.8(11)	37.5(3)	59.3(35)
Gender	Female	43.6 (10)	62.5(5)	49.2(29)
	Male	56.5 (13)	37.5(3)	50.9(30)
Educational level	<Middle school	56.5(13)	50.0(4)	1.7(1)
	completed middle school	30.4(7)	37.5(3)	52.5(31)
	High school (incl. undergrad)	13.0(3)	12.5(1)	42.4(25)
	College/University or above	–	–	3.4 (2)
Hometown	Henan Province	17.4(4)	50.0(4)	13.2(7)
	Anhui Province	34.8(8)	37.5(3)	49.1(26)
	Jiangsu Province	8.7(2)	–	22.6(12)
	Other	39.1(9)	12.5(1)	15.1(8)
Working Status	Student	21.7(5)	–	35.6(21)
	No job, seeking jobs	–	25.0(2)	25.4(15)
	No job, not seeking jobs	–	25.0(2)	1.7(1)
	Full time worker	78.3(18)	50.0(4)	37.3(22)
Average Years Staying in Shanghai (Mean ± SD)		3.1 ± 3.5	3.6 ± 3.5	5.4 ± 6.1
Adults’ Activity				
		KII %(n)		
Gender	Female	85.0(17)		
	Male	15.0(3)		
Occupation	Social worker	15.0(3)		
	Township officer	20.0(4)		
	Neighborhood officer	30.0(6)		
	Community Physician	10.0(2)		
	School health teacher	10.0(2)		
	School head/ideological teacher	10.0(2)		
	Chair of the women’s union in an industrial park	5.0(1)		
Average Years working with migrant youth (Mean ± SD)		6.0 ± 3.5		

Among all the adolescent respondents (*N* = 90), 51.1% were male, and the mean age was 17.4 ± 1.3 years. The average duration they stayed in Shanghai was 4.7 ± 5.4 years. Over three quarters (78.6%) were from the provinces where migrants move out a lot such as Anhui (41.1%), Henan (16.7%), and Jiangsu (15.6%), which was consistent with the data of the sixth national census data. Most of the adolescents (65. 6%) either were studying in middle school, or received under middle school education, or completed middle school education. According to their purpose of immigration, the adolescent respondents could be divided into labor migrants (71.1%) and student migrants (28.9%). Nearly a half (48. 9%) of the youth held a job to earn their living.

Twenty adult key informants (3 male and 17 female) who worked with internal migrant young people in the community were interviewed for about 1 h individually. Because migrant youth could interact with adult services providers in several ways, we purposively selected the key informants from different organizations, which included schools, social work service and psychological counselling centers, neighborhood committees and working places, etc.

### Data analysis

All the activities were audiotaped, transcribed into Chinese, and then translated into English. The interview language was Mandarin because all the migrants can speak and can understand Mandarin fully fluently. Translation was done by two research assistants with fluent English. All translations were double checked by field coordinator to ensure quality. Thematic analysis was approached using ATLAS. Ti 7.0 Software [31, 32]. The analysis included reading, coding, displaying, reducing, and interpreting the text. First, transcriptions were read thoroughly to identify major themes, and then patterns were identified across KIIs, IDIs, PV, and CMGs. Next, codes were developed under the framework of ecological systems theory and acculturation theory as well as issues that emerged from the textual data until the information had been saturated. The major Categories and themes we used were listed in Table 2. Matrices were developed covering broad topics to reduce and summarize the data across groups. Finally, associations across themes were formed, and themes across groups (younger vs. older age group migrant youth, and key informants vs. migrant youth) were compared.

**Table 2:** Major categories, themes and correlated analytic framework targeted

Major categories and themes	Ecological systems theory	Acculturation theory
Understanding of health		
Physical well-being		
Mental well-being		
Social well-being		
Major health issues in host community	Outcomes	Health Outcomes
Mental health		
Smoking		
Violence		
Reproductive health		
Health seeking and host city adaptation	Outcomes	Adaptation Outcomes
Sources of health information		
Health services utilization		
Opportunities (on education/career)		
Factors related to health seeking and city adaptation		Stressors and/or buffers
Economic status Awareness	Microsystem level factors of outcomes	Coping strategies
		Coping resources
Accessibility to community services	Exosystem level factors of outcomes	Social support Social and culture distance
Family, peer support/social network		
Hukou registration status Health insurance Other related policies and laws Social distance and societal attitudes	Macrosystem level factors of outcomes	Societal attitudes

### Ethical considerations

We received permission to carry out the study from Shanghai Institute of Planned Parenthood Research (SIPPR) institute-based ethical review committee (Protocol number #2011–03). We obtained written informed consent for youth equivalent to or above 16 years old and parental/guardian written informed consent as well as youth written assent for participants who were under 16. For key informants, written informed consent were obtained. With regarding to the privacy of image, young participants of PV activity were trained in-depth about how to gain informed consent from the subject or the subject’s parent (for subject who were under 18) for taking his photo and for publication. All photos having individual faces recognizable were signed with written consent [33].

## Results

Our findings first presented how internal migrants perceived “health”, and what they though as the primary health challenges, and then, based on the theoretical framework and related themes came out from the study, we investigated the health seeking behaviors of migrants such as health information acquisition and health service utilization, and their perceived micro-level, exosystem level and macro-level barriers. With regard to city adaptation, a factor influencing both psychological and social well-being of migrants, because opportunities to schooling and job-seeking are essential ways to adapt to the city for internal migrants at 15~17 and 18~19 age groups, respectively, we focused our attention to the structural constrains such as Hukou and insurance policies.

### Young migrants’ understanding of “health”

**Fig. 1:**
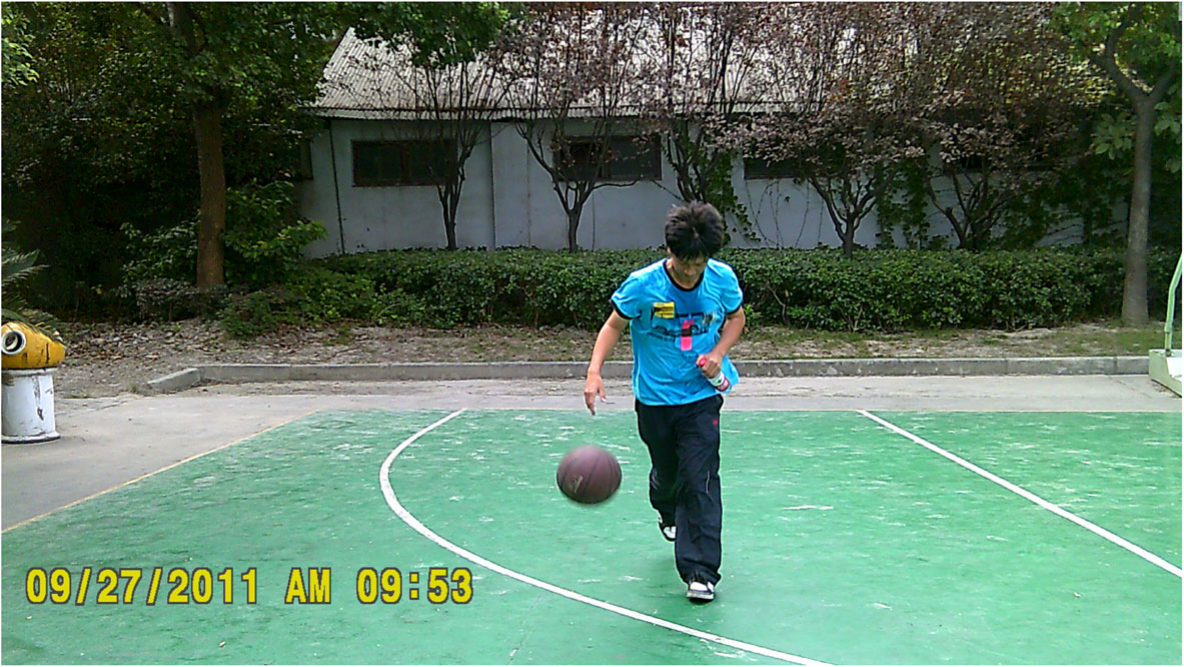
Keep healthy by taking exercise. *“Playing basketball is a healthy lifestyle for adolescents, it can keep you fit, can help you grow taller, it also can help relieve stresses. We should promote such a lifestyle.” (Photographed and explained by a female PV participant of 16 years old.)*

Our study explored young migrants’ understanding of health and found that elder age group youth had a broader concept of health than younger age group youth. In the eyes of the 18~19 year-olds migrant youth, health was not only about phsycial wellness, but also included psychological and social fitness. While in the eyes of 15~17 year-olds migrants, health only meant physically active (see Fig. 1) and not feeling sick; ill health was confined to be a result of a dirty and messy living environment, and the lack of sports and health facilities. Nonetheless, when asking about the importance of health, both groups mentioned the value that “*money could do everything*” was deeply rooted in their mind. They thought their most urgent need to be addressed was the economic problem rather than a health problem at this stage. This kind of thoughts from internal migrant youth was also triangulated by key informants.

*I heard a story told by a community school teacher, he recommended this (a skill learning) project to adolescents one by one in the market, but no migrant youth would want to join in. One said very clearly: “I came to Shanghai to earn money, I couldn’t even feed myself. Now there is a job here to earn money, so I do not want to miss this opportunity, I don’t want to go to school.” (KII, female, social worker).*

### Major health issues in host community

Internal migrant youths and key informants in the study revealed several health issues: One was the mental health problems. Nearly every adolescent talked about frustration, low self-esteem, mood swings and other issues related to mental health when being asked about their prominent health challenges.

*I(Interviewer): If you were to design a program to improve the well-being of floating adolescents in this community, what types of things would be a priority for such a program or would be most useful?*

*A(Adolescent): Mental health.*

*Q: Why? What’s the problem with mental health?*

*A: Because of family things. Life is really hard for migrant parents and it would definitely affect their children.*

*Q: What impact?*

*A: Feel hard and tired. (Choked voice).*

*Q: Are you saying that parents are tired, or kids feel themselves are tired?*

*A: Parents are tired and kids would feel unhappy every time when they see their parents’ fatigue. (Wiping tears) (IDI, female, within 15–17 years old).*

Other health challenges were behavioral related issues. One was their craving for smoking and drinking, especially smoking. They thought *“smoking is very fashionable and cool.” (CMG, female, 18–19 years old group)* One adolescent participant told us: *“I smoke a pack of cigarettes for three days while someone smokes three packs for one day.” (CMG, male, 18–19 years old group)*. In the Photovoice activity, a girl said: *“Precisely to say that migrants have all the problems on the board. If you want me to put them in an order, the first one should be smoking, second is violence, and the third is psychological problems, the fourth is sexual related problems.” (Photovoice, female, within 18–19 years old).* Another issue was related to violence. A key informant (*a female social worker*) said “*Trivial conflicts easily angered migrant youth*”. A youth told us a story: *Once my colleague was skating and he hit a guy unintentionally. Then that guy pretended to get hurt and fall over. He said his head hurt and asked my colleague to take him to hospital. Then many guys environed them, and he said he would kill my colleague if my colleague didn’t take him to hospital. In the end, my colleague gave him 100 Yuan to solve it. (CMG, female, 18–19 years old group).* Therefore, it was not surprising that one of the key informants concluded: *Compared to local young people, there are more migrant youth bullying, brawling and cliquing, which are apt to cause the problem of juvenile delinquency.*

The last but not the least health issue was related to reproductive health. Many migrant youths worked outside unaccompanied, so some of them began cohabitation and had sex unprotected when they were very young, therefore young girls might encounter the problems of unwanted pregnancies. *“The girls sometimes may encounter this problem (abortion) because they are shy and ignorant. Girls don’t know how to refuse the boys.” (KII, female, community doctor).*

### Health seeking behaviors and related barriers

#### Micro-level barriers: constraints from low economic status and services utilization awareness

When asking about health information and services seeking behaviors, 15–17 years old group said they would turn to their parents or teachers for help, while 18–19 years old group would turn to peers or internet. The lack of access to financial and informational support forced migrants to adopt some very unhealthy behaviors when falling ill. They would typically wait and see in the beginning, hoping the illness would go away by itself. Even if they need to go to the hospital to treat the disease, they would take into consideration their money at pocket. Most of them would instead buy medicine from the pharmacies or go to the non-formal clinics. Only when the illness becomes unendurable would they visit hospitals. Due to the financial constraints and social insurance policies, access to large hospitals was a problem for most migrants.

*I haven’t seen any doctors here. I only have the experience of buying medicine from the pharmacy in the community, such as the toothache medicine or pain medication. (IDI, female, within 18–19 years old).*

Besides, most of the migrant youth believed that health risk behaviors, lifestyles or mental health status was a personal matter. They were reluctant to join in the health intervention program (if there were) to change their lifestyles into healthier ones.

Like what migrants perceived, but from a different angle, the key informants thought that the migrant youth had little perception of community resources and services, let alone to make use of those things. They complained that the migrants rarely took the initiative to seek help from service providers.

*I think they don’t have the consciousness of accepting public services. They don’t seek services on their initiative. When you took the initiative to go to them, they would not cooperate, or would not put so much enthusiasm on the activities... They sometimes do not accept or believe us. (KI, female, social worker).*

#### Exosystem level barriers: narrow social network and limited accessibility to community services

**Fig. 2:**
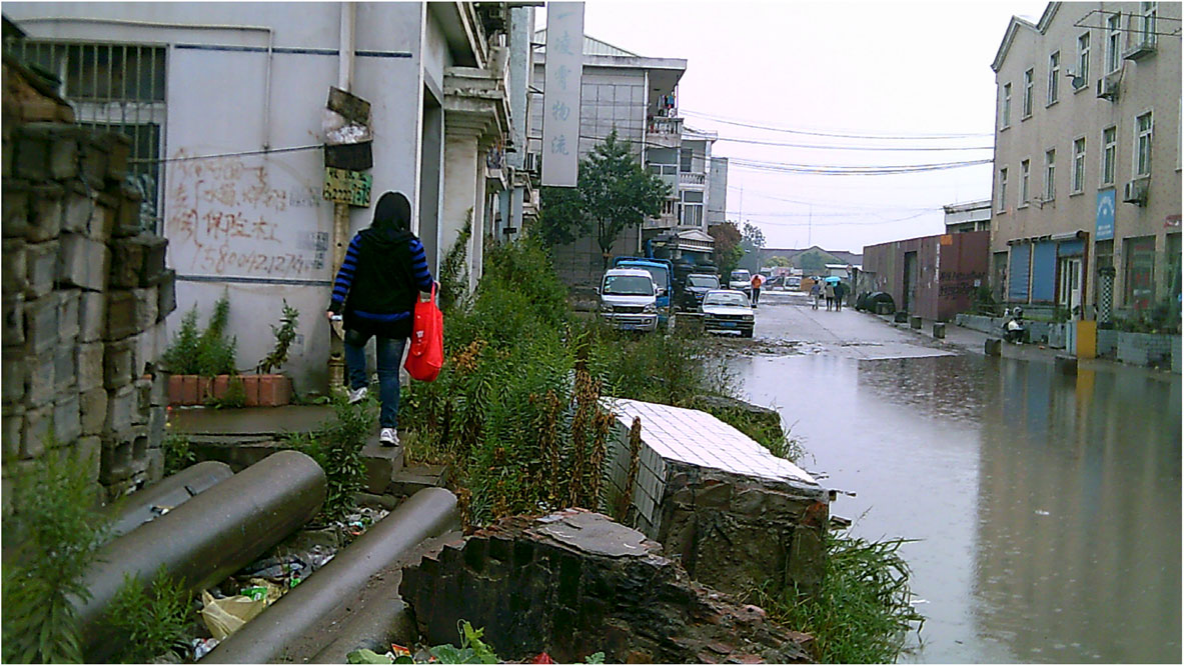
The inconvenience after the rain. *“There is a sunken area without a drainage system besides our neighborhood. It causes inconvenience to the passers-by every time it rains.” (Photographed and explained by a female PV participant of 18 years old.)*

**Fig. 3:**
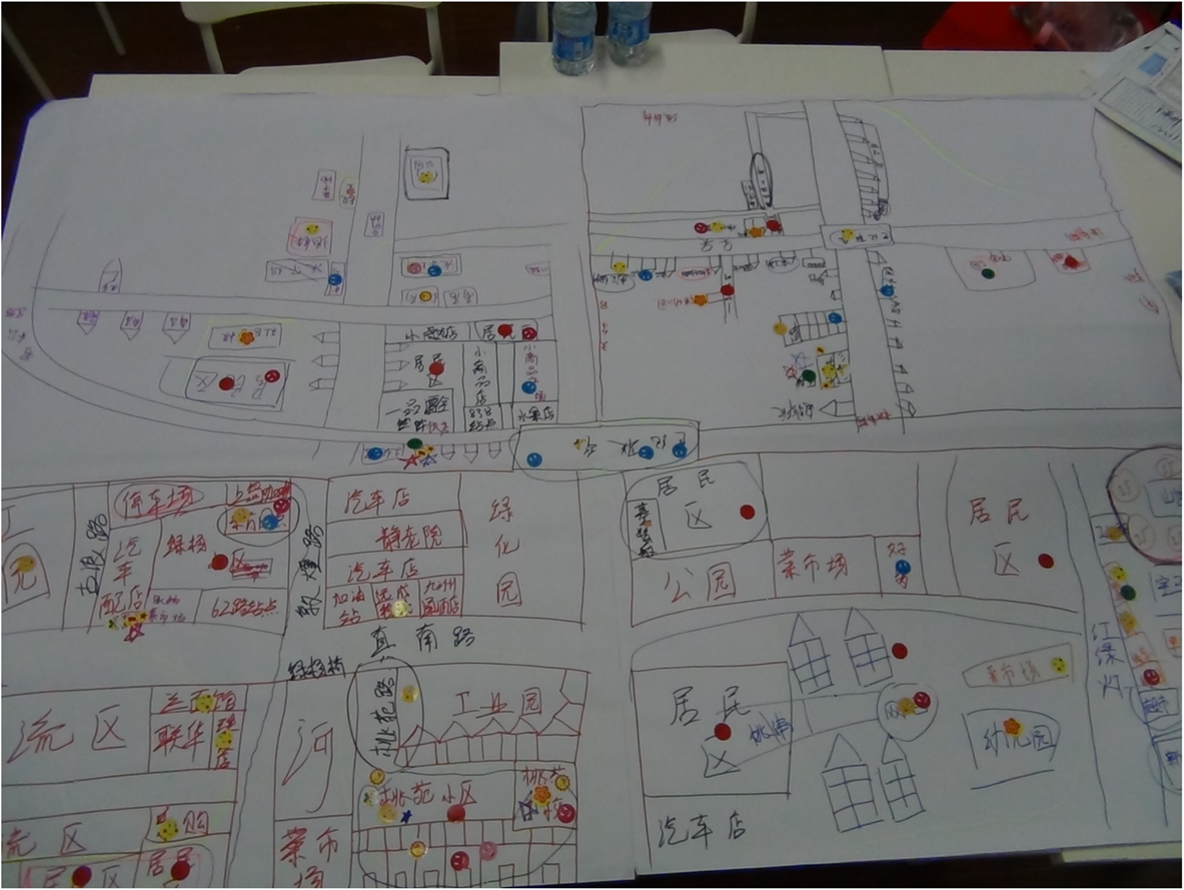
A community map indicating migrants’ living environments and accessible facilities. *The places where internal migrants live concentratedly were drawn on the lower left part of the paper, meaning migrants’ living places were in the fringe area of the community. (Drawn by internal migrant youth of CMG activity, female, 18–19 years old group.)*

Many internal migrants were isolated from mainstream society. Some of them lived with fellow migrants at construction sites, restaurants, or living quarters provided on-site by employers; others lived in the city’s or community’s fringe areas or villages where being dirty, poor, overcrowding, and lack of social and health services (see Fig. 2 and Fig. 3). They were isolated not only in the places of living but also in the aspect of social and cultural interaction. Comparing to labor migrants, student migrants had fixed residence and could get more family support. They considered themselves the same as the Shanghai local students except for having less opportunities to pursue equivalent education. Migrant internal young workers, however, either had fixed residence or not, complained a lot about their living environment during the interview. They mentioned their social interaction in the city did not go beyond that with their relatives or fellow migrants, and suffered the differential treatment with the locals.

While migrant youth perceived the disparity, local key informants confirmed so.

*There are many cars, and it’s noisy and disordered. The road is terrible too. …There is no bus I could take to go to work, and I need to go there by bicycle. …. Besides, there are many thieves, and the public security is not very good.* …*There is no streetlight on the road of our neighborhood (a migrant gathering place). It’s very dark during the night, and it’s terrifying. (CMG, female, 15–17 years old group).*

*The environment is unclean and smelly, like the open sewage. (CMG, female, 18–19 years old group).*

*Most of the migrants live separated from us. We are not in the same neighborhood. The information on job vacancies is (released) from the social insurance center. We will post all the information on the billboard out of our office. The migrants usually do not come here to look for the info, and neither would we go to their living places to deliver this information. (KI, female, neighborhood committee staff).*

#### Macro-level barriers: social distance and societal attitudes towards internal migrants

The migrant youth were afraid of being discriminated against or be given the non-citizen treatment in the process of seeking health information and services. They thought they might necessarily be excluded from the range of service that local organizations and resources provided as they were migrants.

*A few medicines they prescribed would cost you more than three hundred yuan. What was worse, they could not solve your problems. It was not only a waste of my money, but also that evil attitude and humiliation made you very shame of losing faces. And they were not gentle when they did check-ups so that you would feel very hurting. (IDI, female, within 15–17 years old).*

Another health service utilization issue was related to the reproductive health. Most rural-urban migrants were of reproductive and sexually active ages. Due to the social stigma over premarital pregnancy, if a migrant young girl were pregnant, she would turn to the underground clinic for induced abortion; and she would not dare to ask for medical leave from work. Such choices might lead to serious health consequences.

*(...If they got pregnant, they would go to) The underground clinics. They do not want to let others know. …the primary reason might be they don’t want to spread out their shame so that more people will know. But sometimes you need to go to the underground clinics. Everyone knows that the underground clinics are unreliable. (PV, female, within 18–19 years old)*

### Adaptation to host city/community and the structural barriers

#### Schooling opportunities influenced by Hukou status

Though the student migrants considered themselves the same as the Shanghai local students in many aspects, they complained the unequal opportunities of getting further education. Internal migrant students had little access or had to pay a high cost to receive higher educational resources. Because of the hukou registration policies, migrant students found it hard to continue the secondary school education in Shanghai. They were forced to either choose entering vocational schools or returning hometown after graduated from primary school if they wanted to continue learning. The lack of educational opportunities limited their ability for further development.

*For those (migrant youth) who are about the age to go to schools, they are very difficult to be admitted to a school. The school in Shanghai is hard to enter in because of the policy restriction. You almost need to provide over ten certifications from various departments to prove that you are qualified to be admitted to a school. Many schools are not open to migrant youth, which I found the most significant problem facing us. (PV, female, within 18–19 years old).*

#### Working opportunities in the risk of not being protected by health insurance and labor laws

**Fig. 4:**
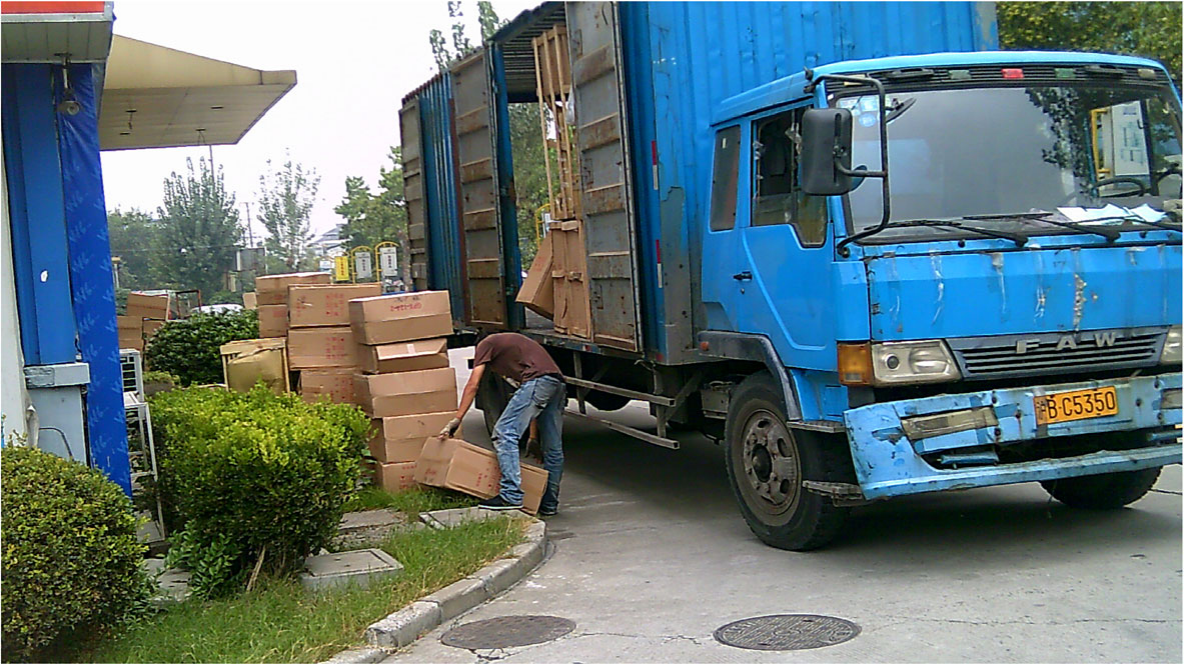
Tired of the work of moving goods. *“He is very young; he is trying to make money by uploading and downloading goods. You could see that the floating young people’s life here is not easy at all. With no skills and poor education, it seems hard for him to get a better job.” (Photographed and explained by a female PV participant of 16 years old.)*

While student migrants complained about unequal access to education, labor migrants complained about unfairness regarding employment opportunities. Most internal migrant young workers we interviewed were on short-term employment contracts or even no contracts at all. They usually undertook low-paying jobs of manual labor in manufacturing, goods transportation, construction, entertainment and restaurant services, with only a deficient percentage of allowance in medical insurance paid by individual enterprises or with no health insurance at all. Those jobs were dirty, tiring, less paid or dangerous so that the locals were not willing to do (see Fig. 4). Migrant workers, especially those who were very young, were very helpless when facing work-related injuries. Enterprises usually treated local (non-migrant) workers much better than did migrants. Most migrant youths didn’t receive any work-skill or self-protection training. If the internal migrant workers did not have labor contracts, they would not have health insurance, and would not be protected by labor laws, their employment situation was precarious.

*We find migrant youth in our age can rarely find a job with enough rest. The position we get is purely relying on our physical work. We repeat the same work day by day. Most of our jobs are monotonous work with less rest time. The basic salary is 1200 per month, and the boss wishes we’d better work as much as 370 days for a year. We are under too much pressure. We are too young to be in the adult world working like this. So, we basically could only work one or two months, and then change to another job. (PV, female, within 18–19 years old).*

*That is a garment factory. The machine is very sharp and operates very quickly. It would even pierce your hand. When you are wounded, you will get a Band-Aid and some disinfectant alcohol to sterilize your skin. Under normal circumstances, they (the employers) won’t take you to the hospital. They directly do the surface sterilization, and then put on a Band-Aid. …I’ve once had my finger pierced by the needle. I bled a lot of blood out. … There was no way to report this kind of thing, and it was useless to say. And there was no one asking for sick leave, because if so, you would lose 150 Yuan from your monthly salary. (PV, female, within 15–17 years old).*

## Discussion

This study was guided by the ecological framework and the acculturation theory as supplementary to explore the young internal migrants’ major health issues, health seeking behaviors and city adaptation from multiple facets. Our findings showed that while younger migrants had a limited understanding of health, a focus on disease and lifestyle dominated the health talk of the youth in the study, elder migrant youths were more sensitive to societal and political factors related to their health. The physical aspects of their settlement were perceived as dirty, mess and poor. Life wasn’t easy if they had no money, even they were young and physically healthy. Mental health and health risk behaviors such as smoking and violence were their major health concerns. Unprotected premarital sex and unintended pregnancy were cited as typical among internal migrant young workers. Key informants reported that internal migrant youths rarely sought health information and services initiatively from formal sources. Though student migrants considered themselves of no difference from the Shanghai locals, they talked about the lack of opportunities to pursue equivalent education. Labor migrants found themselves on the outer stratus of the social sphere in the city, suffering the differential treatment from the locals in working areas. They also talked about lacking social network and supporting policies as the barriers of their health seeking behaviors.

Internal migrant youth in our study cared more about other things than about their health. It was probably attributed to the healthy migrant effect that selectively brings the “brightest and the best” immigrants into host cities. Other studies also suggested that migrant youth showed fewer physical health problems compared to non-immigrant youth despite significant economic disadvantage [16, 34]. Another reason could be that they were at the life stage of youth and they were physically healthy. Comparing to physical health, youth and key informants paid more attention to migrants’ mental health status. A possible explanation was that experiences of discrimination in the acculturation process might be negatively associated with mental health. The migrant youth were reluctant to or even refused to seek help on Sensitive health issues (such as reproductive health), as both migrants and key informants cited, because they were concerned about being discriminated or rejected by others. Frequently being discriminated might create and enhance their perception of social inequity, causing their feelings of inferiority, depression, frustration, and social exclusion, which in turn would result in more psychological problems and less health seeking behaviors.

There are micro-level determinants of health such as income, gender, employment status, as well as exo- and macro-level factors like the sense of community belongings, social support and social economic status. Among which, economic hardship is a significant one and is lined to health disparities [35]. In China, the disparity in disposable income between rural and urban workers is huge. The annual disposable income of urban households in Shanghai (43,851.4 yuan) was the highest among all the regions in 2013 [36], 5.4 times of the net income of rural households (8097.9 yuan) in Anhui province [37]. About 41% of the migrant participants of our study came from rural areas in Anhui provinces. Therefore, it was not difficult to understand that the primary impetus for young migrant workers in our study is to “earn money” to fill the income gap. Lower socioeconomic status could be related to poor living conditions and limited health care accesses. Even they had medical needs, going to hospital would cause substantial economic losses for them with low income. Therefore, underground clinics which provide a relatively small medical fee becomes the solution to the shortage of cheap and convenient medical provision. However, the out of the regulation operation of underground clinics often results in considerable variation in the safety and quality of services and makes the health problems of young people undiscovered. Previous studies indicate that adolescents are more likely to seek abortion services from these unregulated commercial providers due to concerns about inconvenience, fear of disclosure, judgmental attitudes of public sector health workers, and lack of health insurance associated with public hospitals [38]. Access to health care, according to the descriptions from key informants, was also hindered by migrant youths’ lack of knowledge and reachable resources, which was reciprocally influenced by their acculturative stress and the degrees of city adaptation, or to be more explicitly: the stigma of being a “floating youth” in the city. Thus, as a special group in the urban city, adolescent migrants may have greater exposure to health risks but less health seeking behaviors than their peers of local residents.

Whereas native youth typically own a broader range of support providers including more extended family members and friends, migrant youth tend to receive limited support primarily from family members who may or may not live together and peers who migrated together. Our findings on the importance of being socially connected suggested that connections with family and peers had consequential health benefits at individual level. Social networks could be a channel for young migrants to adapt to the host city [39]. For students who immigrate with their parents, interactions in schools can strengthen their social and cultural capital. We saw from our study that the migrant students considered themselves of no difference with the locals. While limited social networks will bring young internal migrants many negative effects on their health, and restrict their healthcare access. In our study, for most of the young migrant workers, their dominant social contacts were the work site interpersonal relationships with colleagues and customers. There social networks were very limited and sometimes unpleasant.

In our study, migrant youth complained about the unsatisfactory housing conditions, the social stigma and discrimination they perceived, and the unequal treatment in seeking jobs and health services compared to urban locals. This is in line with one study conducted in Shanghai, which revealed the great differences of income, work, and social benefits between internal migrant and local workers. Such disparities were mainly caused by the Hukou registration system. For decades, the system had functioned like an “internal passport system” – internal migrants who are not registered in host cities cannot access certain state-level entitlements and social benefits such as free compulsory education, medical care, and employment [40]. Although the Hukou system has been under reform since the 2000s in Shanghai, studies showed that the admittance standards of the reformed policies were still not in the interests of the poor and low-educated migrant workers [41]. They were still excluded from the leading society by being replaced as inferior citizens. In July 2014, the State Council released guidelines on Hukou reform claiming that China would gradually remove the binary rural/urban system and would instead use the name of residence Hukou*.* Hopefully, with the removal of the rural-urban disparity in *hukou*, the education, health insurance, and other welfare attached to *hukou* will be more balanced between migrants and locals in response [17].

Lack of health insurance becomes the prominent stigma for labor migrants in the city [42]. Migrant young workers in our study reported they only had a deficient percentage of allowance paid by medical insurance or even no insurance. The situation is in line with Wang’s study published in 2002: Compared with 79% of local workers owning health insurance, there were only 14% of rural migrants had so [18]. The operational difficulty lies in how to include internal migrants in host city’s medical care system. The legal working contract is critical for the acquisition of health insurance in the host city for immigrants, while the fact is that many migrants enter the job market informally. Though there were studies including migrants into the urban health system in the form of reimbursement of some medical expenses incurred in their cities of work, they were still at an early age [43]. More work needs to be done to remove the structural barriers faced by migrant youth in vulnerable environments, and to evoke migrants to invest time and money in locality or community adaptation.

There are evidences that the availability of support from multiple sources will enhance migrants’ adjustment in the host city [21, 44, 45]. Though family support is important, it normally is not there. In the community and social domain, the relatively narrow network and the sense of helpless could be related to adolescents’ inner and outer problems, such as loneliness, health risk behaviors, and city adaptation [46]. Thus, positive social support from individuals outside the close family circle becomes particularly important to migrants. Several practical actions at community and service levels could be done to improve young internal migrants’ lives in the host city. For example, when new internal migrants come into the cities, the relevant offices in the neighbourhoods could provide information on healthcare related services and where the services are located; schools where there were student migrants and employers of young migrant workers could disseminate pamphlets containing health and career related information and psychological counselling services in their medical clinics (if they have) or in their community. Urban health service workers could visit workplaces and residences with a large proportion of internal migrants to offer education and counselling. Most importantly, the host community need to provide them with services proactively. Policies and more broad services other than healthcare (e.g. Housing, education, job-seeking, insurance, etc.) in relation to the internal young migrants need to reach out to this group of people.

There are certain limitations in this study. First, giving that the management of internal migrants was collaborated by several departments, and family planning department was the major one, we recruited key informants through our contacts in the department of family planning of the township and their network. Although the recommanded key infromants were from different organizations, most of them were not only serving migrants, some of them contacted with migrant youths less frequently than with their local service targets. Making them believed it was the migrants’ fault who had no awareness to turn to local organizations for help. Some of them only worked with certain kinds of migrants, e.g., students, workers, jobless community youths, etc. Therefore, they were only familiar with the types of migrant youths they had served. However, this study benefits from the use of different kinds of qualitative study methods to explore young migrants’ health issue from a multidimensional perspective [30, 47]. More subjective measures to migrant youths were touched, and the perspective of both youths and the community key informants were incorporated. As existing studies often rely on one or the other and there are discrepancies between the two, this combination lessens the bias dramatically. Second, the study is based on findings from fieldwork geographically confined in a migrant community in Shanghai. Therefore generalizability is limited. Nevertheless, it has a potential to seed a lively and constructive debate, in view of the lack of studies on health perception and health-seeking issues of migrant youth. Third, the health problems and influencing factors were quite different among different types of internal migrants, making one solution catering all types of migrants’ interest impossible. In our study we saw disparities among labor migrants and student migrants. Further comparative studies should be done regarding their types of migration such as whether they were brought by their parents, came and joined others, or migrated by themselves. Given the sparse knowledge of migrant youths’ perception of health risks and health service utilizing, it is necessary to conduct further studies to consider their interests, and of the community health services capacity as well.

## Conclusions

The study’s findings provide the insight to the social contexts which influence the health experience, health seeking behaviors and city adaptation of young internal migrants, and found that mental health and related health risk behaviors, such as smoking, violence, sexual and reproductive health problems were their primary health challenges, most internal migrant youth were marginalized in the host city. They could make use of few health service resources, had fewer opportunities on education and career, and perceived more social stigmas and inequalities. Lack of awareness, social support and supporting policies, as well as the acculturative stress--the stigma of being a “floating youth”, impeded their health-seeking behaviors and city adaptation. Our study implies the importance of understanding social relationships and structural barriers faced by migrant youths in vulnerable environments. The result also indicates that multidimensional social support is essential for young migrants facing potential health risks.

## Additional files

Additional file 1: KIIs Guide. Key Informant Interview Guide. (DOCX 23 kb)

Additional file 2: IDIs Guide. Youth In-Depth Interview Guide. (DOCX 23 kb)

Additional file 3: CMG Guide. Community Mapping and Focus Group Field Guide. (DOCX 22 kb)

Additional file 4: PV Guide. Photovoice Project Guide. (DOCX 23 kb)

## Abbreviations

CMG: Community mapping & focus group discussion
IDI: In-depth Interview
KII: Key informant interview
PV: PhotoVoice
SIPPR: Shanghai Institute of Planned Parenthood Research
WAVE: Well-being of Adolescents in Vulnerable Environments
