# Supplementary material for: Young internal migrants’ major health issues and health seeking barriers in Shanghai, China: a qualitative study
Source: BMC Public Health. 2019 Mar 22;19:336. doi: 10.1186/s12889-019-6661-0 (PMC6431074; doi:10.1186/s12889-019-6661-0)
Supplement: Supplementary file 1 — KIIs Guide. Key Informant Interview Guide. (DOCX 23 kb) [file 12889_2019_6661_MOESM1_ESM.docx]

KEY INFORMANT INTERVIEW GUIDE

***Thank you for taking the time to talk with me today about your understanding of floating youth’s health in this community. I would like to ask you a few questions before we get started.***

**BACKGROUND INFORMATION**

Mark Gender of Participant: ___Male ___Female

How long have you worked with youth in this community? ____ years (#)

***Let’s get started with our discussion.***

1. Can you tell me about your organization? Where does your work take place?
2. How would you describe the floating youth you serve?

- Probe: Are the youth you serve typical of this community?
- Probe: How are they different?
- Probe: How are they similar?

1. What are some of your challenges in serving floating young people here? How do you usually deal with these challenges?
2. In addition to your organization, what other resources exist for floating young people in the community?

- Probe: What types of direct health services exist? Community and youth development programs?
- Probe: Services related to violence? Substance use? Mental health? Sexual/reproductive health such as consultation, health education material delivering, contraception providing and abortion operation providing?

1. What do you see as the primary challenges facing floating youth in this community? Have you taken any measures to deal with these challenges? (If yes) Are there any effects? Are there any deficiency? (If no) Are there any difficulties?

- Probe: Is it different for boys vs. girls? How?
- Probe: Is it different for older youth vs. younger youth? How?

1. What are the primary health issues for floating youth? Have you taken any measures to promote their health? (If yes) Are there any effects? Is there any deficiency? (If no) Are there any difficulties?

- Probe: How much of a problem is violence (e.g. fighting / being abused)? Substance use? Mental health (e.g. feeling vacant / lonely / nervous / anxious / depressed and suicidal)? Sexual/reproductive health (e.g. relationship issues / unprotected sex / STIs / pregnancy / abortion)?
- Probe: Is it different for boys vs. girls? How?
- Probe: Is it different for older youth vs. younger youth? How?

1. Where do floating adolescents go for health information (e.g., medical healthcare info and reproductive health knowledge on contraception, pregnancy, STIs / AIDS, etc.)? What is the difference between the floating youth and local youth in getting the health information above?

- Probe: What is the role of friends and family? Working place? Community? School? Internet? Formal services (e.g. hospital or family planning service station)?
- Probe: Is it different for boys vs. girls? How?
- Probe: Is it different for older youth vs. younger youth? How?

1. Where do they go for health services (e.g. See the doctor / Go for a health consultant / Get a prescription of drugs or buy drugs)? (Sexual/reproductive health such as getting some consultation / contraception / abortion services? Mental health? Substance use? Violence?) As far as you know, how about the service quality? Credibility? Privacy protection? What is the difference between the floating youth and local youth in acquiring the above services?

- Probe: What is the role of friends and family? Working place? Community? School? Internet? Formal services (e.g. hospital or family planning service station)?
- Probe: Is it different for boys vs. girls? How?
- Probe: Is it different for older youth vs. younger youth? How?

1. What are some of the barriers floating youth face when seeking health information and services?

- Probe: Are there policies that might restrict certain services from being offered to floating youth? If so, could you explain?
- Probe: How convenient are the locations for floating youth in this community?
- Probe: What about the hours of operation? Are services/information available after school/on weekends?
- Probe: How informed are floating youth about the types of health information/services that exist here?
- Probe: Are there any types of services/information that are needed for floating youth in this community, but don’t exist? If so, could you describe them? How do some of these barriers vary for girls and boys? How might they vary by age of the youth?

***Thank you again for your time.***
