# Supplementary material for: Young internal migrants’ major health issues and health seeking barriers in Shanghai, China: a qualitative study
Source: BMC Public Health. 2019 Mar 22;19:336. doi: 10.1186/s12889-019-6661-0 (PMC6431074; doi:10.1186/s12889-019-6661-0)
Supplement: Supplementary file 2 — IDIs Guide. Youth In-Depth Interview Guide. (DOCX 23 kb) [file 12889_2019_6661_MOESM2_ESM.docx]

YOUTH IN-DEPTH INTERVIEW GUIDE

***Thank you for agreeing to speak with me today. I am interested in hearing more about you, your experiences living in __________________ (name of community), and how those experiences have influenced your health and well-being.***

***Before we get started with our chat, I would like to ask you just a few basic questions.***

**BACKGROUND INFORMATION**

1. Mark Gender of Participant: ___Male ___Female
2. How old are you? ____ years (#)
3. What is the highest grade of school you have completed?

__None or some primary

__Primary complete

__Some junior high school

__Junior high school complete

__Some senior high school

__Senior high school complete or above

1. Do you currently earn money? ___Yes ___No
2. If so, what do you do to earn money? ________________________________________
3. Where you born in this neighborhood (from where recruited)? ___Yes ___No
4. If not, where were you born? _______________________________________________

(document country & city/neighborhood)

1. a. How long have you been in Shanghai? _______years_______months

b. How long have you lived in this community? _______years_______months

***Now let’s get started with our conversation. Please remember I am very interested in hearing from you and getting to know what it’s like to live in ________from your perspective. Just to remind you, everything you share stays between you and I, so please be as open as possible.***

- - 1. Please start by telling me what a typical week-day is like for you?
- Probes: What do you do? Where do you go? How long do you work/study in a typical week-day? Who do you see?
  - 1. What about on the weekends, what is a typical weekend like for you?
- Probes: What do you do? Where do you go? Who do you see? What do you do for fun?
  - 1. Tell me a little bit about what it is like living in ___________ (community name)?
- Probes: What do you like about living in _____? What don’t you like about living in_____?
- Probes: Have you always lived in this area? If not, where did you live previously? What brought you and your family here?
- Probes: How about your relationship with other people who live in this community? Are you familiar with your neighborhood? Do you get along well with them? Do you have communication with them?
  - 1. How does living in ___________(community name) affect the way people treat you?
- Probes: Have you ever felt or been discriminated against because you are from this community? Have you ever felt or been discriminated against because you are a migrant?
  - 1. What is it like for the floating adolescents living in this community (name)? What is different from local adolescents?
- Probes: What is different for floating adolescents e.g. what is more challenging or easier for them with regard to living in the community?
  - 1. How is it different being a girl from this community vs. a boy from this community?
- Probes: daily affairs? access to health services? legal help? entertainment? etc.
- Probes: What is different for girl vs. boy adolescent e.g. distinct challenges for girls or boys?
  - 1. Speaking now about you in particular, tell me about your goals for the future…
- Probe: What do you see yourself doing in five years: e.g. studying, job, family?
- Probe: What types of support systems or help do you feel you need to reach these goals?
  - 1. What types of things are you personally currently most worried about overall?
- Probes: What would you say are your most pressing problems? What’s on your mind a lot? Why?
  - 1. Where do you go for help about these concerns and issues?
- Probes: What types of organizations or people do you turn to? How do they help?
  - 1. If you were designing a program to improve the well-being of floating adolescents in this community, what types of things would be a priority for such a program or be most useful?
    2. What about your health? Where does that fit in, in terms of the things that you are concerned about or spend time thinking about?
    3. Are there particular things that concern you regarding your health and well-being?
- Probes: Tell me more about those issues…How did you deal with it? Have you ever sought for health care service or help? What was the result?
- Probes: Violence, mental health and substance use, sexual and reproductive health
  - 1. Tell me about your last experience seeking health care services…
- Probes: When was the last time you sought health care services? (document how long ago)
- Probes: Type of services? Where sought? How did you feel about these services? Is it convenient? How is the attitude of service provider? Cost? Was the problem solved?
- Probes: What types of barriers did you face in terms of accessing these services? If you did face some barriers, was it because you were a migrant?
  - 1. Have you ever been to community health service center for health care?
- Probe: If yes, tell me about your experience (s) getting health care at community health service center.
- Probe: If no, tell me about why you generally don’t seek care at community health service center.
  - 1. Have you ever needed help for a health problem, but couldn’t get it?
- Probe: Tell me more about that…What happened? What did you do? What do you think the main reason is? Is it because you are a migrant? How was the problem solved?
  - 1. In terms of health information or health related materials, please tell me about the most recent type of health information you have received
- Probes: When was the last time you received any health information or materials?
- Probes: Types of messages/materials? From where or who received?
- Probes: Were these messages/materials useful, how or how not so?
  - 1. What type of health information do you feel like you need, but don’t currently have? Where would you like to get the information? In which way?
- Probe: Violence prevention or care services
- Probe: Mental health services and support
- Probe: Substance use e.g. alcohol and drug use
- Probe: Sexual and reproductive health, HIV/AIDS

***Thanks very much for your time and sharing your thoughts and experiences.***
