# Supplementary material for: Young internal migrants’ major health issues and health seeking barriers in Shanghai, China: a qualitative study
Source: BMC Public Health. 2019 Mar 22;19:336. doi: 10.1186/s12889-019-6661-0 (PMC6431074; doi:10.1186/s12889-019-6661-0)
Supplement: Supplementary file 3 — CMG Guide. Community Mapping and Focus Group Field Guide. (DOCX 22 kb) [file 12889_2019_6661_MOESM3_ESM.docx]

COMMUNITY MAPPING AND FOCUS GROUP FIELD GUIDE

*“Hello. We are from* Shanghai Institute of Planned Parenthood Research *working with Johns Hopkins School of Public Health and AstraZeneca on a study about floating adolescents in this community and how you find and use health information and services. For this day, we are first going to be asking you all to draw what you think is your “community.” After you draw your community, we’ll be asking you a few more questions about it, where in the community you go for help, support, and services, and then we’ll take a break, and ask you a few more questions about some other aspects of our study…*

*Does anyone have any questions for me at this time?*

***[Mapping]***

- Tell the group to put different stickers on their map to indicate where the homes and residential areas, the schools, the places where youth hang out and other places they interested (e.g., shops, cyber cafe, etc.) are in their community. Ask the indicated places one by one:

*“Can you tell me more about these homes? How would you describe them? Do floating adolescent stay in one place? How often might someone your age moves from different residence?”*

*“Can you show me where you put your stickers? What are these schools? Do any of you attend these schools?”*

*“Could you tell me about these places? What are the reasons why youth go to these places? What kinds of things do youth do at these places?”*

*“Could you tell me what you put down for this sticker? Are these popular places for young people your age? What are the reasons why young people would visit them?”*

- Have youth indicate on their map where they would go if they wanted to receive information or help on each specific information and service:
  - 1. Information about pregnancy prevention
    2. Treatment for a sexually transmitted infection
    3. A place to get condoms or contraceptives
    4. Help about violence in the family/other types of violence
    5. Help or services for getting a job
    6. Help or services for stress or depression
    7. A place to use computer/surf the net
    8. Help or services for drugs or alcohol

For each place, facilitators can use the following set of questions to collect more details about these places.

- If stickers were placed inside community:

1. Tell me about these places (Probe: Is it a clinic? A school? Etc.)
2. How do you know about this place?
3. How well does this place offer SERVICE to adolescents?
4. Are there other places outside this community that would be better? If so, what are they?

- If stickers were not placed in inside community:

1. Where could floating adolescents go to get information and services about this?
2. How would floating adolescents from this community go there?
3. What are some of the reasons why floating adolescents would go to this place for this service?

***[Focus Group discussion Guide]***

***“Okay, now let’s look at our maps with all of the features pointed out…”***

The community context of adolescent health information & services:

1. Can you “walk” me through your community and tell me a little more about what you put down on your map? [Have them point these out.]
2. How would you describe your community to outsiders?
   1. What do you like best about it?
   2. What don’t you like about it?
3. How long have you lived in this community?
   1. [If haven’t lived in community entire time after they came to Shanghai, probe where they’ve lived and why they think they moved to this community]
4. If you could change one thing about this community, what would it be?
   1. What are the reasons why you would change it?
5. Where on this map are the places you all feel safest during the day? Can you circle those with yellow?
   1. Could you tell me more about what makes you feel safe there?
   2. What types of things are you safe from?
6. What about during the night, where on this map do you feel safest? (can you circle those places in blue)?
   1. Could you tell me more about what makes you feel safe there?
   2. What types of things are you safe from?
7. What about the places where you don’t feel safe during the day? Could you circle those places in red?
8. What are the reasons why you don’t feel safe there?
9. Where on this map are the places where you don’t feel safe during the night? Can you circle those places in black?
   1. What are the reasons why you don’t feel safe there?
10. How ‘healthy’ would you say this community is for floating adolescents?
    1. What are the factors that influence the health of floating adolescents in this community?
       1. What about the family – how do families influence the health of floating adolescents?
       2. How do schools?
       3. How does this community?
       4. How do peers?
11. What are things that this community needs for floating adolescents to be healthy?
    1. Tell me why these things help floating adolescents be healthy?
12. In this community, what is the best place for getting health information? Can you put a blue star to indicate this on your maps?
    1. What makes this place the best?
    2. What type of health information can you get there?
    3. How do floating adolescents know about this place?
    4. What are the reasons that would prevent some floating adolescents from using this place?
    5. Are there places outside this community that are better for getting health information?
       1. If so, could you tell me about those places?
13. What about health services in general? Where in this community is the best place for receiving health services? Can you put a red star to indicate this on your maps?
    1. What makes this place the best?
    2. What type of health services can you get there?
    3. How do floating adolescents know about this place?
    4. What are the reasons that would prevent some floating adolescents from using this place?
    5. Are there places outside this community that are better for getting health services?
       1. If so, could you tell me about those places?

*“That is great. Does anyone have any questions about anything we discussed?*

Before the end, facilitators should take pictures of each map.

*“Thank you!”*
