# Supplementary material for: Young internal migrants’ major health issues and health seeking barriers in Shanghai, China: a qualitative study
Source: BMC Public Health. 2019 Mar 22;19:336. doi: 10.1186/s12889-019-6661-0 (PMC6431074; doi:10.1186/s12889-019-6661-0)
Supplement: Supplementary file 4 — PV Guide. Photovoice Project Guide. (DOCX 23 kb) [file 12889_2019_6661_MOESM4_ESM.docx]

PHOTOVOICE PROJECT GUIDE

**DAY ONE**

- Administrative Business
  - Researchers and photographers review the agenda for the four day project
  - Participants review and sign the Photovoice Project Activity Acceptance Form
  - Distribute cameras and notebooks
- Photographer leads an Introduction to photography
  - Introduce photos as a visual language using examples of photography
  - Share past photovoice projects
  - Introduce the fundamentals of storytelling with photos
  - Introduce participants to the cameras and instruct them in the basics of working the cameras
  - Introduce participants to captioning and recording each photo they take in a log
    - Discuss photo release form
    - Review ethics of having photo release signed
    - How to take photos of people without showing faces
    - Discuss the responsibility of using a camera
- Practice taking photos in the community
  - Specific photo assignments are given
- Return to download photos and review the photos taken
- Download photos from the practice session
  - Teach participants how to download the photos from the cameras
  - Participants select photos for discussion in small groups
- Using the SHOWed method, conduct a structured discussion of the photographs. Participants will be divided into two small groups for the discussion of the photographs using the questions below. Participants will be drafting captions for their photographs as part of the discussion. Themes from these discussions will be recorded on flip charts and the groups will rejoin to discuss the themes that surfaced in each discussion group.
  - What do you See here?
  - What is really Happening?
  - How does this relate to Our lives?
  - Why does this problem or strength exist?
  - What can we Do about it?
    - Questions to supplement the SHOWed method (that may be more acceptable for youth):
      - What are your reasons for taking a photo of this?
      - What does this photo demonstrate about your community?
      - What story does it tell?
- Introduce the Photovoice theme of Health and *Well-Being in our Community*. The one overarching question to be answered through Photovoice is, “What does health and well-being mean to you?”
  - Stress a focus on the well-being of young people and what it means to be an adolescent

If young people are in need of clarification, researchers may provide the WHO definition of health, *Health is a state of complete physical, mental, and social well-being and not merely the absence of disease or infirmity* as well as provide broad examples*.* However researchers will strive to allow the youth participants to define health and well-being.

---

**DAY TWO**

- Administrative business
  - Students arrive, check-in and talk about any problems/successes they had. Make sure cameras are working properly and notebooks, pens and releases are in hand.
- Go out into the community to take photos that tell a story about Health and *Well-Being in our Community*
- Download photos from community photo session
- Select photos for discussion in small groups
  - Ask the following questions:
    - What do you see here?
    - What are your reasons for taking a photo of this?
    - How does this photo help you to define ‘Health and Well-Being’?
    - What story does it tell about Health and Well-being in our community?
- Themes from these discussions will be recorded on flip charts and the groups will rejoin to discuss the themes that surfaced in each discussion group. The following questions may be used as prompts during the theme discussion.
  - What are the main threats to health and well-being that face floating youth in the community?
    - How do young people define health and well-being?
    - How do young people know when their health and well-being is threatened?
    - How do young people maintain good health and well-being?
    - How do young people restore their health and well-being when they face a problem?
    - What can we do to improve the health and well-being of young people in this community?
- Lunch
- Go out into the community to take photos that tell a story about Health and *Well-Being in our Community*
- Download photos from community photo session
- Select photos for discussion in small groups
  - Ask the following questions:
    - What do you see here?
    - What are your reasons for taking a photo of this?
    - How does this photo help you to define ‘Health and Well-Being’?
    - What story does it tell about Health and Well-Being in our community?
- Themes from these discussions will be recorded on flip charts and the groups will rejoin to discuss the themes that surfaced in each discussion group. The following questions may be used as prompts during the theme discussion.
  - What are the main threats to health and well-being that face floating youth in the community?
    - How do young people define health and well-being?
    - How do young people know when their health and well-being is threatened?
    - How do young people maintain good health and well-being?
    - How do young people restore their health and well-being when they face a problem?
    - What can we do to improve the health and well-being of young people in this community?

---

**DAY THREE**

- Administrative business
  - Students arrive, check-in and talk about any problems/successes they had. Make sure cameras are working properly and notebooks, pens and releases are in hand.
- Go out into the community to take photos that tell a story about Health and *Well-Being in our Community*
- Download photos from community photo session
- Select photos for discussion in small groups
  - Ask the following questions:
    - What do you see here?
    - What are your reasons for taking a photo of this?
    - How does this photo help you to define ‘Health and Well-Being’?
    - What story does it tell about Health Well-Being in our community?
- Themes from these discussions will be recorded on flip charts and the groups will rejoin to discuss the themes that surfaced in each discussion group. The following questions may be used as prompts during the theme discussion.
  - What are the main threats to health and well-being that face floating youth in the community?
    - How do young people define health and well-being?
    - How do young people know when their health and well-being is threatened?
    - How do young people maintain good health and well-being?
    - How do young people restore their health and well-being when they face a problem?
    - What can we do to improve the health and well-being of young people in this community?
- Lunch
- Go out into the community to take photos that tell a story about Health and *Well-Being in our Community*
- Download photos from community photo session
- Select photos for discussion in small groups
  - Ask the following questions:
    - What do you see here?
    - What are your reasons for taking a photo of this?
    - How does this photo help you to define ‘Health and Well-Being’?
    - What story does it tell about Health and Well-Being in our community?
- Themes from these discussions will be recorded on flip charts and the groups will rejoin to discuss the themes that surfaced in each discussion group. The following questions may be used as prompts during the theme discussion.
  - What are the main threats to health and well-being that face floating youth in the community?
    - How do young people define health and well-being?
    - How do young people know when their health and well-being is threatened?
    - How do young people maintain good health and well-being?
    - How do young people restore their health and well-being when they face a problem?
    - What can we do to improve the health and well-being of young people in this community?

---

**DAY FOUR**

- Administrative business
  - Students arrive, check-in and talk about any problems/successes they had. Make sure cameras are working properly and notebooks, pens and releases are in hand.
- Go out into the community to take photos that tell a story about Health and*Well-Being in our Community*
- Download photos from community photo session
- Select photos for discussion in small groups
  - Ask the following questions:
    - What do you see here?
    - What are your reasons for taking a photo of this?
    - How does this photo help you to define ‘Health and Well-Being’?
    - What story does it tell about Health and Well-Being in our community?
- Themes from these discussions will be recorded on flip charts and the groups will rejoin to discuss the themes that surfaced in each discussion group. The following questions may be used as prompts during the theme discussion.
  - What are the main threats to health and well-Being that face floating youth in the community?
    - How do young people define health and well-Being?
    - How do young people know when their health and well-Being is threatened?
    - How do young people maintain good health and well-being?
    - How do young people restore their health and well-being when they face a problem?
    - What can we do to improve the health and well-being of young people in this community?
- Lunch
- Each participant will work with the photographer and researcher to select their top ten photos for enlargement and refine the captions for those photos
  - Finalize release forms
